# Effect Sizes for 2×2 Contingency Tables

**DOI:** 10.1371/journal.pone.0058777

**Published:** 2013-03-07

**Authors:** Jake Olivier, Melanie L. Bell

**Affiliations:** 1 School of Mathematics and Statistics, University of New South Wales, Sydney, Australia; 2 Psycho-Oncology Co-Operative Research Group, School of Psychology, University of Sydney, Sydney, Australia; University of East Piedmont, Italy

## Abstract

Sample size calculations are an important part of research to balance the use of resources and to avoid undue harm to participants. Effect sizes are an integral part of these calculations and meaningful values are often unknown to the researcher. General recommendations for effect sizes have been proposed for several commonly used statistical procedures. For the analysis of 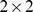 tables, recommendations have been given for the correlation coefficient 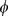 for binary data; however, it is well known that 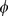 suffers from poor statistical properties. The odds ratio is not problematic, although recommendations based on objective reasoning do not exist. This paper proposes odds ratio recommendations that are anchored to 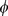 for fixed marginal probabilities. It will further be demonstrated that the marginal assumptions can be relaxed resulting in more general results.

## Introduction

Sample size calculations are an integral part of scientifically useful and ethical research [1]. A study which is too small may not answer the research question, wasting resources and potentially putting participants at risk for no purpose [2]. Studies which are too large can also waste resources and expose participants to the potential harms of research needlessly, as well as delaying results and their translation into practice. The computation of sample size *a priori* is usually dependent upon predetermined values for power and level of significance, an estimate of the expected variability in the sample and an effect size of practical or clinical importance. By convention, the choice of power and level of significance is usually at least 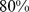 and no more than 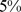 respectively. When a practically important effect size is unknown, there are several recommendations in the literature to guide the researcher. In his seminal paper, Cohen [3] gives operationally defined small, medium and large effect sizes for various, common significance tests. The use of effect size recommendations should not replace differences of clinical or practical importance [4] and may not be appropriate for all disciplines. In basic science research, for example, large effect sizes by Cohen's criteria are common and, therefore, require small sample sizes. On the other hand, clinical and epidemiological research often deals with small effect sizes and often requires large, population-based studies. While there are some approaches to estimating a minimum important effect [5], there are instances where this information is simply not known. Thus, effect size recommendations assist with the balance between overly small and overly large sample sizes.

When the researcher is interested in 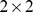 contingency tables, a common measure of effect size is 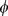 which, in this instance, is equivalent to Pearson's correlation coefficient [6]. Cohen [3] recommends effect sizes of 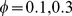 and 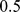 for small, medium and large effect sizes respectively and are identical to his recommendations for the correlation coefficient. Although Cohen [3] denotes this statistic as 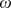, much of the literature uses 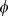 [6]–[10] and the remainder of this manuscript follows this convention. To support his recommended effect sizes for correlation coefficients, Cohen [11] chose equivalent values for the difference in two means through the connection with point biserial correlation. Additionally, 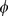 is applicable to logistic regression since it can be converted to an odds ratio (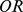) when the row (or column) marginal probabilities of the 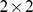 table are fixed. For example, when the marginal probabilities are uniform (i.e., 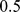 for row and column probabilities), Cohen's recommended effect sizes are equivalent to odds ratios of 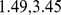 and 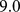. It will be demonstrated that the connection between the odds ratio and 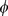 is largely dependent on the marginal probabilities and these 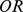 values should not be used in general.

A problem arises when using the effect size 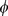 for 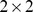 tables as the full range of correlation coefficients are only possible under very restrictive circumstances and are not justified in general [12]. On the other hand, odds ratios are valid effect size measures that are not constrained by the marginal probabilities. Ferguson [10] recommends small, medium, and large odds ratio effect sizes of 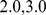 and 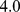, but urges caution in their use as they are not “anchored” to Pearson's correlation coefficient. Although many have pointed out problems with 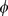 as an association measure and advocate the use of odds ratios as an alternative, effect size recommendations for odds ratios do not exist in general.

It is common in randomised controlled trials and case-control studies to fix one of the marginal probabilities in the 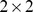 table as it directly relates to the ratio of participant allocation. For instance, a marginal probability of 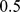 corresponds to a 1:1 case-control ratio while a 2:1 ratio is a marginal probability of 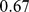 (or equivalently 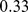 for 1:2).

The aims of this paper are to demonstrate: (1) the equivalence of effect size measures for 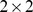 contingency tables, in particular the relationship between 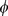 and the odds ratio; (2) that recommended odds ratio effect sizes can be derived from Cohen's work using the maximum value of 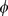 as a guideline for fixed marginal probabilities; (3) the shortcomings of 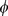 and the strength of the odds ratio as an effect size measure; and (4) that conservative odds ratio effect size recommendations can be derived without relying on fixed margins. We provide an example that investigates the association between helmet wearing by bicyclists and overtaking distance by automobiles.

## Equivalence of Effect Size Measures for 2×2 Contingency Tables

### 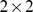 Contingency tables

The two-way classification or contingency table is a common method for summarising the relationship between two binary variables, say 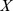 and 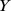. Table 1 gives the joint probability distribution of 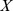 and 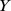 when their individual outcomes are from the set 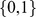.

**Table 1:** 2×2contingency table of probabilities.

	*X = *0	*X = *1	*Total*
*Y = *0	π_00_	π_01_	π_0+_
*Y = *1	π_10_	π_11_	π_1+_
*Total*	π_+0_	π_+1_	1.0

In this formulation, 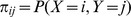, for 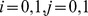, is the joint probability of 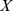 and 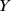, 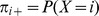 is the marginal probability of 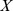, and 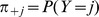 is the marginal probability of 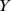. Under an assumption of independence between 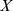 and 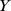, the product of the marginal probabilities equals the cell probabilities, i.e., 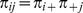. Alternatively, the 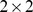 table could be represented by the frequency of observations so that 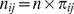 where 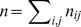. Similarly, the marginal frequencies are 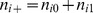 and 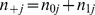. Note that 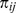 is assumed to be the population proportion as the focus of this paper is the use of effect sizes as a planning tool and not statistical inference per se. In a case-control study, for example, 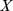 may indicate the presence or absence of disease while 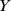 is an indication of exposure. Thus, 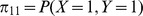 would represent the joint probability of being diseased and exposed.

### Effect size 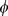 and Equivalences for 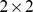 Tables

There are many association measures applicable to 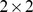 tables which, with the exception of the odds ratio and relative risk, are equivalent or similar to 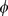. The equivalence of some of these association measures is outlined below.

For the random sample 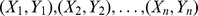, Pearson's correlation coefficient is
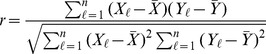
where 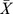 and 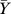 are the sample means of the 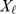 and 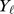 respectively. Although used primarily as a measure of linear association, Pearson's correlation coefficient can be applied to binary variables and is often given the notation 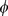. For the 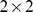 table case, we get



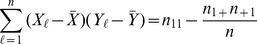


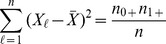


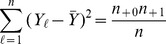



So, Pearson's correlation coefficient for binary random variables 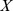 and 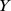 is
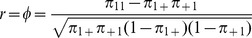



Since 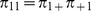 under the hypothesis of independence, 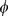 can be interpreted as measuring the departure from independence between 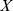 and 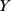. Note that Cramér's 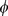 is equivalent to this equation for the 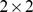 table case [11] as well as the square root of Goodman and Kruskal's 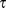 [13].

For the analysis of contingency tables, in general (not just the 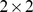 table case) the effect size formula for 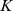 total cells is
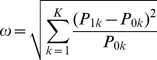
where 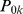 and 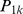 are cell probabilities under the null and alternative hypotheses respectively. Note that 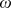 is related to the usual chi-square statistic 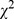 by 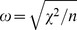 and is sometimes called the contingency coefficient. Using this formula, Cohen [3] recommends 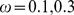 and 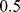 for small, medium and large effect sizes. Making note that 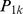 is the probability of each cell (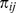) and 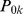 is the cell probability under an independence assumption (so that 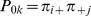), we can then write the effect size formula for the 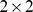 table as follows



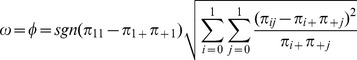



Simple arithmetic demonstrates the equivalence of 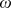 with 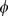. The 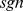 function is used to give the appropriate sign since the chi-square statistic is inherently non-directional.

### The relationship of 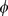 to the odds ratio

The odds ratio for the association between 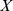 and 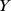 is 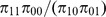. When the marginal probabilities are held constant and the cell probability 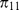 is known, the remaining cell probabilities can be written as
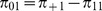


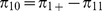


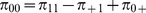



Therefore, when the marginal probabilities are fixed, the odds ratio can be computed directly from 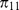, which can then be expressed as
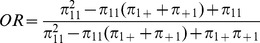



It is clear from the above formula that the odds ratio will be greater than one (or less than one) precisely when the joint probability 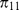 is greater (or less) than expected under an assumption of independence, i.e., 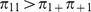. Additionally, the formula for 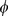 can be rearranged to solve for 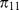, i.e.,
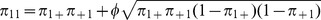



Although mathematically unattractive, it is clear the odds ratio can then be computed from 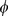, 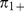, and 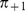. Note that when 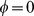 (i.e., no correlation), we get 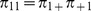 (i.e., 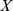 and 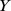 are independent) and the odds ratio is 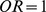. When 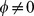, the term 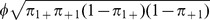 is then a measure of the departure from independence.

## Maximum 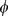 and Modified Effect Sizes

When the marginal probabilities are fixed constants, 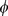 is an increasing linear function of 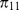. Further, 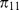 is bounded by
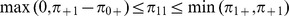



These bounds are due to all cell probabilities being non-negative and the relationship of 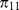 with the other cell probabilities given above. As a result, 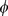 is bounded as well and attains its maximum when 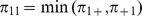. Using the upper bound of the above inequality, it can be shown that
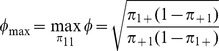
where 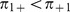 to ensure 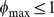. It is clear from the formula for 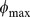 that the full range of correlation coefficients, i.e., 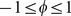, is attainable only when the marginal probabilities are equal, i.e., 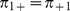 or 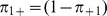. This has an intuitive appeal as perfect correlation for two binary variables is only possible when two cell probabilities are zero. For example, when all observations are in either the 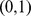 or 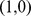 cells, 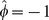. However, it would appear highly unlikely both marginal probabilities will be equal in practice. For example, in a 1:1 case-control study with mortality as the primary outcome, half of all patients would need to die for perfect correlation to be possible. On the other hand, if 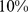 of all patients die, the maximum correlation possible is 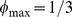 which is near a medium recommended effect size. So, in this situation, all estimates of 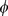, computed from observed proportions, are bounded by



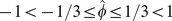



**Figure 1:**
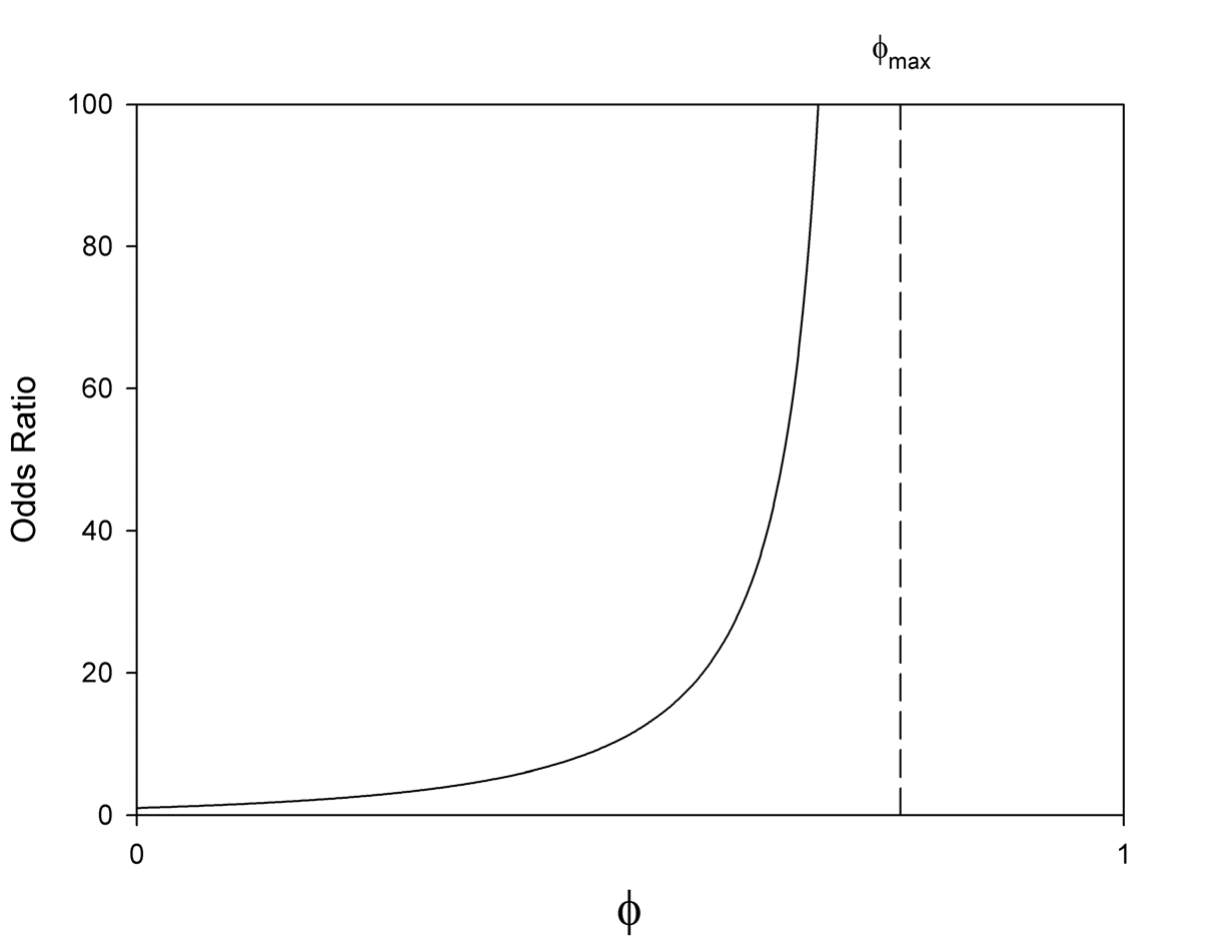
Relationship between the odds ratio and 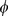 for unequal marginal probabilities.

Importantly, odds ratios are not bounded with possible values of 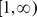 as 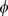 varies on the interval 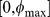. In fact, as 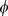 approaches 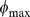, the 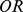 increases without bound. Figure 1 demonstrates this relationship. Importantly, this indicates 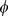 has serious limitations as a measure of association and that these limitations are not applicable to the odds ratio.

## Effect Sizes Relative to 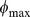


In many practical instances, the marginal probabilities are not equal, making the full range of values for 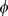 impossible with the potential of making Cohen's recommended effect sizes unusable for 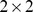 tables. Although not equivalent to perfect correlation, 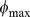 can be interpreted as the maximum possible correlation given the marginal probabilities. In fact, 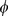/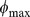 has been proposed as an association measure with the interpretation as the proportion of observed correlation relative to the maximum attainable with fixed marginal probabilities [7], although the researcher is cautioned when the marginal probabilities diverge [6]. Note that 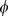 is not equivalent to Cohen's similarity/agreement measure 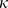. However, 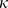 suffers from the same boundary problems as 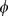 and the two are equivalent when scaled to their maximum values, i.e., 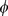/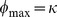/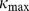, making the two measures similar [6].

### Recommended effect sizes in terms of the odds ratio

**Figure 2:**
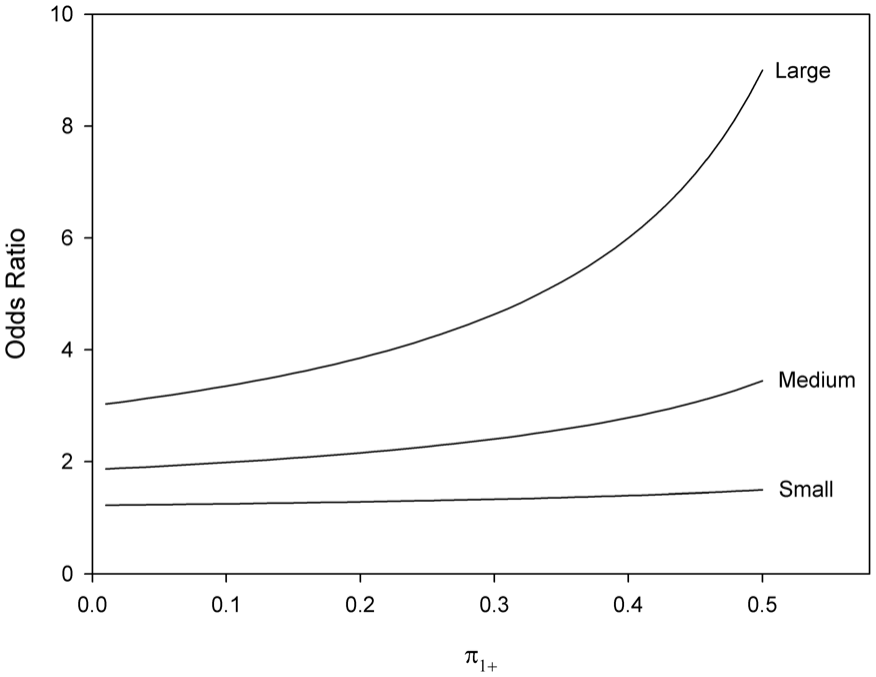
Odds ratios and marginal probability by small, medium and large effect sizes for 1:1 allocation.

**Figure 3:**
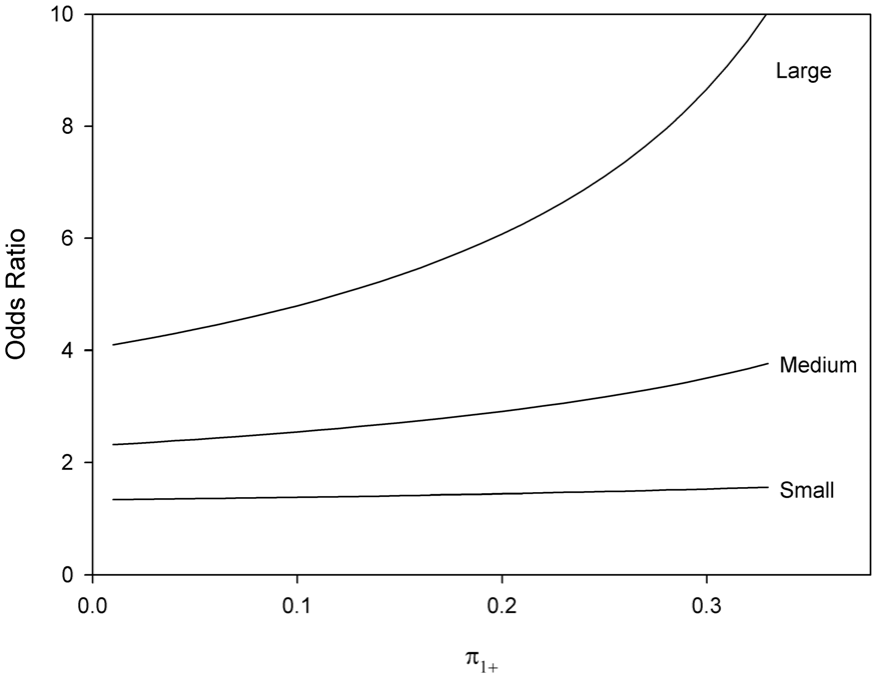
Odds ratios and marginal probability by small, medium and large effect sizes for 1:2 allocation.

As an alternative to Cohen's recommendations, increments of 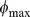 can be related to the odds ratio, say 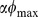, where 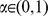. Note that values of 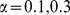 or 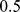 coincide with Cohen's usual recommendations when 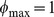. The relationship between 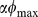 and the odds ratio can be simplified by choosing marginal probabilities for commonly used participant allocations. As an example, Figures 2 and 3 demonstrate the relationship between 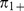 and odds ratios for 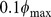, 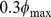 and 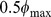 for 1:1 and 1:2 allocations respectively. Note that the minimal odds ratios, and therefore most conservative when used to compute sample size, occur when 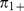 tends to 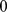. Although the odds ratio does not exist when 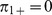, the limit exists and is
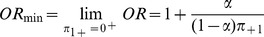



Additionally, the maximal odds ratio, and therefore most anti-conservative, occurs when the marginal probabilities are equal, as expected. Below is the maximum attainable odds ratio for equal margins 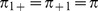 for increments 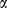 of 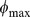,
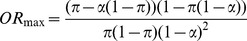



It is important to note that when 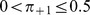, as is often true for case-control studies where cases are harder to identify or enrol than controls, the minimal odds ratio will be smallest for evenly allocated studies, i.e., 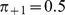. Further, it is generally recommended to use 1:1 allocation as it is the most statistically efficient ratio, i.e., maximum power for a fixed overall sample size. So, odds ratios of 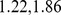 and 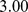 can be used as small, medium and large effect sizes without assumptions regarding marginal probabilities. Sample sizes computed using these odds ratios for 1:1 allocation are given in Table 2 for 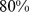 power and 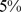 level of significance. A SAS macro that will compute sample sizes from given marginal probabilities for small, medium and large odds ratios has been provided as a supplementary file.

**Table 2:** Sample sizes calculated for small, medium and large effect sizes for 1:1 allocation, 80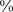 power and 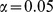.

					π_1+_				
Odds Ratio	0.1	0.2	0.3	0.4	0.5	0.6	0.7	0.8	0.9
1.22	8168	4688	3646	3254	3188	3386	3948	5282	9576
1.86	724	436	354	330	338	374	454	632	1188
3.00	200	128	110	108	116	134	170	246	480

Interestingly, Haddock et al. [12] as a rule of thumb consider odds ratios greater than 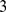 large effect sizes, although there is no clear justification given. In a situation where an allocation ratio other than 1:1 is used, recommended odds ratios can be computed directly using the above formula. These results are also applicable for other values of 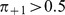 through its complement 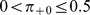. This is equivalent to swapping the columns (or rows) and the researcher should be aware the recommended odds ratio effect sizes are now the reciprocals of those above, i.e., 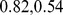 and 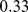 for small, medium and large respectively.

This approach can also be applied to the relative risk and risk difference. If 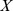 is taken as the grouping variable and 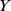 as the outcome, the relative risk is 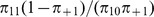. Simple substitution of 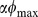 and the marginal probabilities 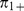 and 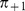 results in a relative risk identical to 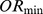 for 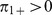, i.e.,
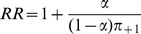



Therefore, recommendations can also be derived for relative risk and are identical to those given for the odds ratio above. This result is expected as the odds ratio converges to the relative risk as the incidence rate approaches 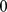.

Instead of comparing the risk between two groups as a ratio, it is sometimes useful to compare their differences [14]. Again taking 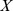 as the grouping variable and 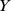 as the outcome, the risk difference can be written as
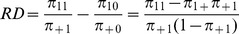



where 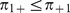 to ensure 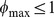 as above. It is clear from the numerator in this representation that 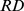 is a measure of the departure from independence, i.e., 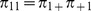. Simple substitution of 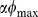 into 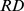 yields
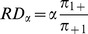
where the subscript 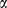 is used to distinguish between risk difference formulae. This formula can be simplified somewhat for 1:1 allocations, i.e., 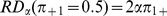; however, a general result independent of the marginal probabilities is clearly not possible in this instance as 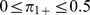 and therefore 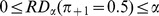.

Alternatively, the 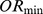 formula can be solved for 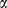 and compared to previously given odds ratio recommendations. In terms of 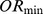 and 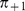, we get
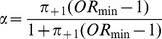



When the allocation ratio is 1:1, this formula simplifies to 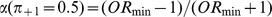 which has a form identical to Yule's 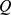 [15]. So, Ferguson's [10] odds ratio recommendations of 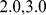 and 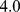 therefore correspond to proportions of maximum correlation of 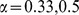 and 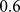. This suggests Ferguson's recommendations have the potential to be anti-conservative from a sample size viewpoint.

## Example

This paper was motivated by a reanalysis of passing distances for motor vehicles overtaking a bicyclist [16]. One of the primary results of this study was a significant association between helmet wearing and less overtaking distance, supporting a theory of risk perception for motor vehicle drivers directed towards bicyclists. Prior to collecting data, Walker [16] reported computing a sample size of 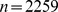 overtaking manoeuvres based on a 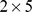 fixed effects factorial ANOVA for a small effect size 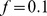, 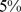 level of significance and 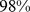 power. The factors for this study were helmet wearing (2 levels) and bicycle position relative to the kerb (5 levels). It has been noted, however, that passing distances are often recommended and sometimes legislated to one metre or more [17]. So, passing manoeuvres of at least a metre are considered safe and less than a metre unsafe, with the implication that large differences in passing distance are unimportant beyond one metre in terms of bicycle safety. When compared with helmet wearing, safe/unsafe passing distances can be analysed using a 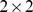 table. Since Walker's study was powered at an unusually high level with subsequent increased probability of a type I error, bootstrap standard errors were estimated for more reasonable values for power of 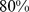, 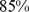 and 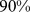. Operationally defined small, medium and large effect sizes were also used since a meaningful difference in overtaking distance is unknown.

The relevant observed data from Walker [16] is given in Table 3. The observed marginal proportions here are 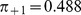 for helmet wearing and 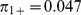 for unsafe passing manoeuvres. Using the marginal probabilities, the maximum attainable effect size is 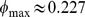 and the estimated correlation is 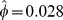. A consequence is the effect size for the association between helmet wearing and safe passing distance is, at best, much less than a small effect size by Cohen's index. The corresponding small, medium and large odds ratio effect sizes using increments of 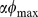 are 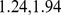 and 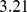 for 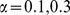 and 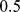. Note that these values are not much greater than the minimal recommended odds ratios mentioned in the previous section, further suggesting the association between safe/unsafe passing distance and helmet wearing is, at best, a small effect size. In fact, the unadjusted odds ratio is 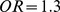 and non-significant by the chi-square test (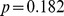). Conversely, sample sizes for a future study can be computed from the observed probabilities using G*Power for logistic regression with a single binomially distributed predictor for 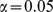 and 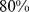 power [18] resulting in 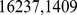 and 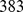 observations for small, medium and large odds ratios. To put these sample size computations into perspective, a future study would need to extend the sampling period by a factor greater than seven to detect a significant association between helmet wearing and safe/unsafe overtaking distance given a small effect size and identical marginal probabilities.

**Table 3:** Observed proportion of helmet use and safe passing manoeuvres from Walker (2007).

	No Helmet	Helmet	Total
Safe	0.491	0.462	0.953
Unsafe	0.021	0.026	0.047
Total	0.512	0.488	

## Discussion

We present a demonstration that many contingency table correlation measures are equivalent for the 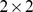 case and their use is limited due to constraints created by fixed marginal probabilities. The odds ratio, which is a function of these measures for fixed marginal probabilities, is not problematic, is regularly used in statistical analyses and has a direct application to logistic regression. Recommended odds ratios have been proposed from Cohen's small, medium and large effect sizes for 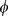 relative to the maximum attainable correlation 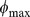. Further, minimal odds ratios can be computed with only knowledge of participant allocation.

The use of effect size recommendations should be avoided in situations in which clinical or practical differences are known. However, they can help the researcher balance between overly large or overly small sample size calculations when such information is unknown. In these situations, conservative estimates for odds ratio effect sizes can be derived from only the allocation ratio leading to a general result and, when a 1:1 allocation is chosen for optimal power, odds ratios of 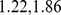 and 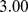 correspond to small, medium and large effect sizes.

## Supporting Information

File S1 **SAS Macro to compute sample sizes from marginal probabilities for small, medium and large odds ratios.**
(SAS)
